# Epigenetic Regulation of Ferroptosis-Associated Genes and Its Implication in Cancer Therapy

**DOI:** 10.3389/fonc.2022.771870

**Published:** 2022-01-31

**Authors:** Yanzi Pei, Yujie Qian, Hao Wang, Li Tan

**Affiliations:** ^1^ Center for Medical Research and Innovation, Shanghai Pudong Hospital, Fudan University Pudong Medical Center, and Shanghai Key Laboratory of Medical Epigenetics, Institutes of Biomedical Sciences, Fudan University, Shanghai, China; ^2^ Department of Anesthesiology, Zhongshan Hospital, Fudan University, Shanghai, China

**Keywords:** ferroptosis, epigenetic regulation, cancer, DNA methylation, histone modifications, RNA methylation

## Abstract

Ferroptosis is an evolutionarily conserved form of regulated cell death triggered by iron-dependent phospholipid peroxidation. Ferroptosis contributes to the maintenance of tissue homeostasis under physiological conditions while its aberration is tightly connected with lots of pathophysiological processes such as acute tissue injury, chronic degenerative disease, and tumorigenesis. Epigenetic regulation controls chromatin structure and gene expression by writing/reading/erasing the covalent modifications on DNA, histone, and RNA, without altering the DNA sequence. Accumulating evidences suggest that epigenetic regulation is involved in the determination of cellular vulnerability to ferroptosis. Here, we summarize the recent advances on the epigenetic mechanisms that control the expression of ferroptosis-associated genes and thereby the ferroptosis process. Moreover, the potential value of epigenetic drugs in targeting or synergizing ferroptosis during cancer therapy is also discussed.

## Introduction

Ferroptosis, which was first proposed by Dixon et al. in 2012, is a novel programmed cell death driven by iron-dependent accumulation of lipid peroxidation (1–7). As an evolutionally-conserved cell death form, ferroptosis plays a critical role in diverse physiological and pathological processes (8). Apparently, understanding the comprehensive molecular mechanisms of ferroptosis has great biological importance and clinical significance. Epigenetic regulation controls gene expression by writing/reading/erasing the covalent modifications on DNA, histone, and RNA, without altering the DNA sequence (9). Accumulating evidence suggests that epigenetic regulation modulates the expression dosages of ferroptosis-associated genes and consequently contributes to the determination of cell sensitivity to ferroptosis. Here we summarize current knowledge on the role of epigenetic regulation in ferroptosis and its implication in cancer therapy.

## The Regulatory Circuits of Ferroptosis

### Biochemistry of Ferroptosis

Since the ferroptosis was first defined in 2012, the core biochemical components of ferroptosis have been rapidly identified in the past decade.

#### Lipid Peroxidation

Polyunsaturated fatty acids are susceptible to lipid peroxidation and essential for the ferroptosis process (10). The abundance and localization of polyunsaturated fatty acids determine the level of intracellular lipid peroxidation and furtherly decide the sensitivity of cells to ferroptosis (11). Phosphatidylethanolamines (PEs) containing arachidonic acid are the major phospholipid that peroxides and promotes ferroptosis (7, 12). Further on, the PE-Coenzyme-A derivatives form and insert into phospholipid, which was defined as a necessary step of pro-ferroptotic signal production (7). In 2015, through the haploid genetic screening, Dixon et al. identified 9 lipid metabolism-associated genes, including the lipid remodeling gene *LPCAT3* and fat acid metabolism gene *ACSL4*, that play essential roles in ferroptosis (5). Doll et al. convincingly proved that ACSL4 is involved in the generation of pro-ferroptotic state (7).

#### Intracellular Iron

Iron complexes or loosely bound iron structure are essential for the formation of reactive oxygen (13). In eukaryotic cells, the uncoordinated redox-active Fe^2+^ that was temporally released in the plasma is generally referred as “The Labile Iron Pool” or “free Fe^2+^” (14). Technically, free Fe^2+^ catalyzes the formation of hydroxyl radical and hydroxide from H_2_O_2_ through “Fenton reaction”. Consistent to the name of “ferroptosis”, both Fenton reaction and iron-dependent enzymes are the formation of reactive oxygen, which causes severe oxidative damage to neighbor cell structure, are key players in ferroptosis (15, 16). Conversely, treatment with iron chelators such as could inhibit ferroptosis. Under normal physiological condition, the level of iron is well regulated by transferrin (extracellular environment) and ferritin (intracellular environment). Apparently, the signaling pathways that alter iron metabolism have potential effect on the regulation of ferroptosis.

#### Glutathione and GPX4

**Figure 1:**
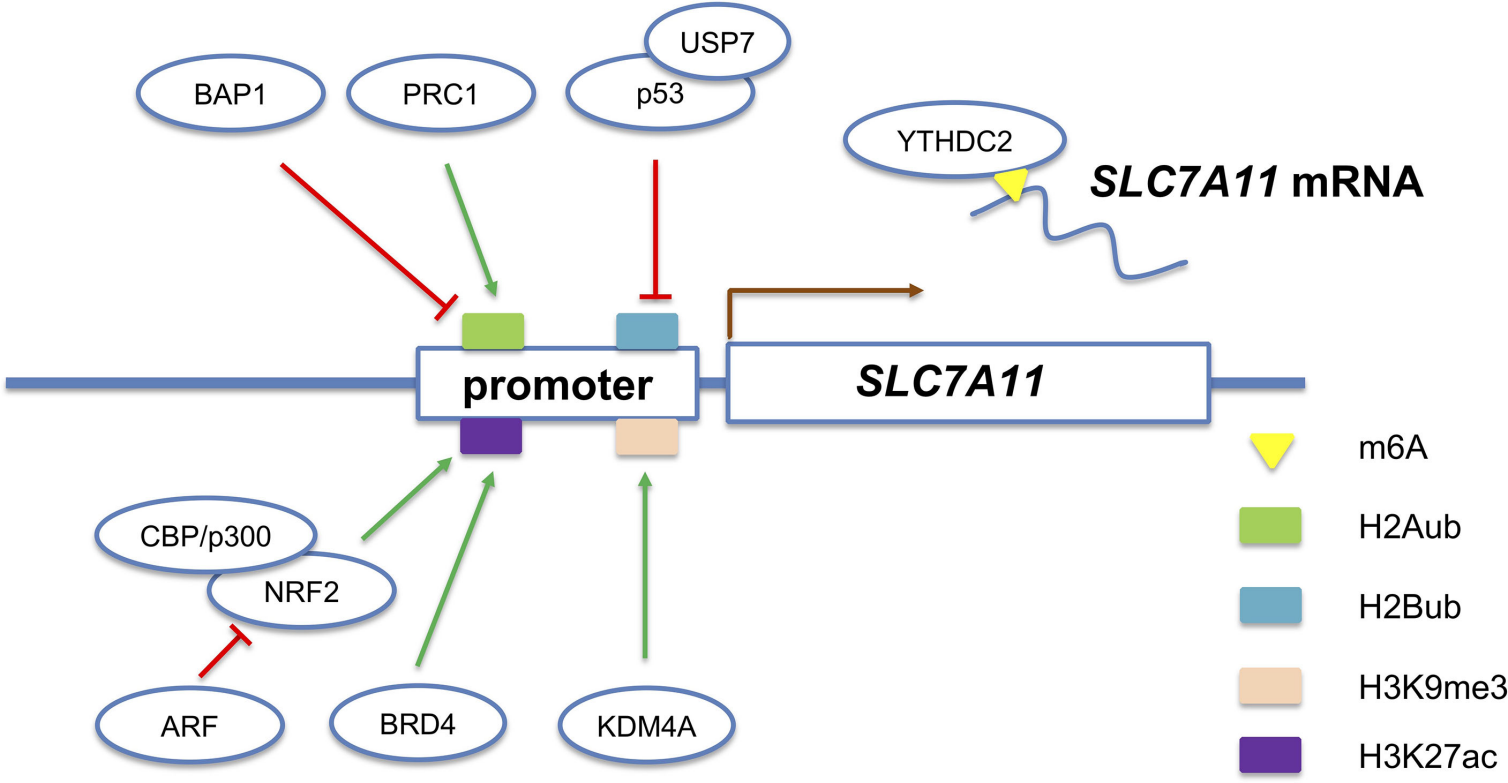
Multiple epigenetic mechanisms regulate the expression of *SLC7A11* gene, a representative ferroptosis-associated gene.

Glutathione (GSH) metabolism was identified as the first pathway regulating the ferroptosis process. The hydro-sulfuryl structure makes GSH as a commonly considered antioxidant and free radical scavenger in the intracellular environment (17). In 1986, the glutamate-cystine transportation system $X_{\text{c}}^{-}$ was identified by Bannai et al. (18). The transporter is composed by one light chain subunit and one heavy chain subunit, which were respectively encoded by *SLC7A11* and *SLC3A2*. System $X_{\text{c}}^{-}$ transfers glutamate out of cells and cystine into cells at a ratio of 1:1, and then cystine is reduced into cysteine which participates in the synthesis of GSH (**Figure 1**) (19). High concentration of extracellular glutamate inhibits cystine uptake through inhibition of Xc^-^ leading to glutathione decrease and oxidative cell death (20, 21). Indeed, the first ferroptosis inducer, erastin, mainly targets on system $X_{\text{c}}^{-}$ (1).

The glutathione peroxidases (GPXs) are series of peroxide-degrading enzymes. GPX4 uses GSH as an essential cofactor to prevent lipid peroxide and maintain redox homeostasis (22). In 2008, Seiler et al. identified lipid peroxidation as the key mediator of cell death in glutathione peroxidase 4 (GPX4) knockout cells (23). Thus, people began to consider this type of cell death different from either apoptosis or necrosis. Several small molecule compounds were screened out as ferroptosis inducer (24–27). Among them, RSL3 targets on GPX4 (25).

#### The FSP1-CoQ10 Pathway

Independent of GPX4 regulatory pathway, the FSP1-CoQ pathway is a novel ferroptosis pathway identified by Doll et al. through an expression cloning approach to identify genes in human cancer cells that are able to complement the loss of GPX4 (6).

Doll et al. revealed that apoptosis-inducing factor mitochondria-associated 2 (AIFM2, renamed as FSP1) overexpression can largely abrogate GPX4 inhibition-induced ferroptosis. A previous study showed that FSP1 functions as a NADP dependent coenzyme Q (CoQ) oxidoreductase *in vitro* (28). CoQ_10_ is a mobile lipophilic electron carrier that endogenously synthesizes lipid-soluble antioxidants and acts as a lipophilic free radical-trapping agents (RTAs) in the plasma membrane (29). Intriguingly, FSP1 overexpression fails to suppress ferroptosis in both CoQ_2_ knockout cells and in cells treated with the CoQ_2_ inhibitor (6, 30). CoQ_2_ is the enzyme that catalyzes the first step in CoQ_10_ biosynthesis, and the soluble analog of CoQ_10_ is sufficient for suppressing ferroptosis and lipid peroxidation (6, 30). These two latest studies clearly suggest that FSP1 acts parallel to GPX4 to inhibit ferroptosis by regulating the nonmitochondrial CoQ_10_ antioxidant system.

#### The DHODH-CoQH_2_ Pathway

Dihydroorotate dehydrogenase (DHODH), an enzyme essential for the *de novo* biosynthesis of pyrimidine-based nucleotides, is a known therapeutic target for multiple diseases (31). Furthermore, DHODH inhibitors, including brequinar, leflunomide, and teriflunomide, have been clinically evaluated but failed to receive FDA approval for the treatment of cancer (32–34). Mao et al. identified DHODH as the third anti-ferroptosis pathway, independent of GPX4 and FSP1 (35). Mechanistically, DHODH in the mitochondrial inner membrane regulates the production of CoQH_2_, a radical-trapping antioxidant in mitochondrial. Importantly, DHODH inhibitor brequinar selectively suppresses GPX4-low tumor growth by inducing ferroptosis, whereas combined treatment with brequinar and sulfasalazine synergistically induces ferroptosis and suppresses GPX4-high tumor growth.

#### The GCH1-BH4 Pathway

Tetrahydrobiopterin (BH4) is a redox-active cofactor involved in the production of nitric oxide, neurotransmitters, and aromatic amino acids (36). The GCH1-PTS-SPR pathway catalyzes GTP to BH4, and GCH1 is a rate-limiting enzyme in the synthesis of BH4 (37, 38).

Kraft et al. found that the overexpression of GCH1 provide protection to against ferroptosis by abolishing lipid peroxidation (36). GCH1 overexpression exhibits robust protection against RSL3- and imidazole ketone erastin (IKE)-induced ferroptosis and genetic ablation of *GPX4*-induced ferroptosis but does not protect cells against inducers of apoptosis and is only marginally effective against necroptosis. Those results indicate that GCH1 selectively inhibits ferroptotic cell death (36).

Intriguingly, BH4 loss in cells leads the accumulation of coenzyme A, NADP, and oxidized GSH (GSSG) in cells. Further, the elevation of reduced CoQ_10_ in cells with GCH1 overexpression have been detected (38). Thus, these results indicate that the GCH1-BH4 pathway acts as an endogenous antioxidant pathway to inhibit ferroptosis through a mechanism independent of the GPX4 signal pathway.

### The Signal Pathways of Ferroptosis

Several canonical oncogenic and tumor suppressive pathways have been reported to converge to the ferroptosis process. In general, these pathways alter the ferroptosis sensitivity through modulating the expression levels and enzymatic activities of core ferroptosis executors.

#### The p53 Pathway

The p53 pathway inhibits cystine uptake and sensitize cells to ferroptosis through repressing SLC7A11 expression (39). Notably, the acetylation–defective mutant p53^KR^ loses the function of inducing cell-cycle arrest but still retains the ability to regulate SLC7A11. Moreover, the spermidine/spermine N1-acetyltransferase 1 (*SAT1*) gene, which encodes a rate-limiting enzyme in polyamine catabolism, was identified as a transcription target of p53 and promote ferroptosis through conversing spermidine and spermine back to putrescine (40). However, there were also some controversial reports on the function of p53 pathway in ferroptosis. For instance, the dipeptidyl-peptidase-4 (DPP4) can be blocked by p53, resulting in resistance to ferroptosis (41). Alternatively, the wild-type p53 stabilization can delay the induction of ferroptosis in cancer cells upon system $X_{\text{c}}^{-}$ inhibition (42). These findings indicate a content-dependent role of p53 in the regulation of ferroptosis.

#### The KEAP1-NRF2 Pathway

It has been well-established that nuclear factor erythroid 2-related factor 2 (NRF2) pathway plays an essential role in antioxidant response. The correlation between NRF2 pathway and ferroptosis has also been studied (43, 44). NRF2 upregulates system $X_{\text{c}}^{-}$ and thereby protects brain tumor cells from ferroptosis (45). Since the NRF2 pathway is commonly activated in diverse malignant tumors, it is likely that aberrant NRF2 activation contributes to protect tumor cells against ferroptosis. A recent work revealed that 3D organoid culture causes ferroptosis and insufficient NRF2 activation leads to the failure of establishment of organoids (46). In addition, the tumor suppressor ARF (CDKN2A) has recently been identified as a binding partner for NRF2 and impacts ferroptosis sensitivity (47). Mechanistically, ARF represses NRF2-induced transcriptional upregulation of SLC7A11 and other antioxidant genes.

#### The Hippo Pathway

Hippo pathway controls organ size by regulating cell proliferation, apoptosis, and stem cell self-renewal (48). Wu et al. observed that high cell density protects many types of cells against ferroptosis during the *in vitro* cell culture. Furthermore, they revealed that the cell density-dependent acquisition of ferroptosis resistance is triggered by E-cadherin-mediated activation of intracellular NF2 (also known as merlin) and Hippo signaling pathway (49, 50). Antagonizing this signaling allows the nuclear translocation of proto-oncogenic transcriptional co-activator YAP and promotes ferroptosis by upregulating several ferroptosis modulators, including ACSL4 and TFRC (50). The identification of E-Cad/Hippo/YAP/ACSL4 axis may explain a long term-observed phenotype that mesenchymal cells are more sensitive to ferroptosis than epithelial cells (50). Similarly, epithelial-mesenchymal transition (EMT) increases the vulnerability of cells to ferroptosis, which may be partially attributed to the inactivation of Hippo pathway during EMT (51).

Besides the above three well-established signaling pathways, other canonical development and disease-associated pathways such as RAS-RAF-MAPK and PI3K/Akt also have intersection with the core regulatory circuity of ferroptosis and participate in the regulation of ferroptosis process.

## Epigenetic Mechanisms Underlying Ferroptosis

### DNA Modification

DNA methylation is the most common epigenetic modification that has been studied in gene regulation. Homocysteine treatment induced DNA methylation of *GPX4* gene in nucleus pulposus, leading to ferroptosis sensitivity (52). DNA hypermethylation of *CDH1* gene promoter in head and neck cancer cells repressed E-cadherin (encoding by *CDH1*) expression and increased ferroptosis susceptibility (51). These two cases clearly demonstrate that DNA methylation is involved in the epigenetic silencing of ferroptosis-associated genes. However, whether other ferroptosis-associated genes are also affected by DNA methylation requires further study. Moreover, TET (ten-eleven translocation) proteins could catalyze 5mC oxidation, which in turn initiates the active or passive DNA demethylation (53). It is still unclear whether TET proteins-mediated DNA demethylation also plays a role in the regulation of ferroptosis.

### Histone Modifications

Histones form the framework of DNA entangling (54). The tails of four core histones (H2A, H2B, H3 and H4) are proved to undergo chemical modifications, including lysine methylation/acetylation, arginine methylation/citrullination and serine/threonine/tyrosine phosphorylation (55). These chemical modifications alter the interaction between histones and other nuclear proteins including the transcriptional machine, thereby changing the expression of targeted genes.

Histone acetylation marks are written by histone acetyltransferases (HATs), read by bromodomains (BRDs), and erased by histone deacetylases (HDACs). NRF2 activates the transcription of *SLC7A11* gene partially through recruiting HATs (CBP and p300) (47, 56). Moreover, the expression of many ferroptosis-associated genes (*GPX4, SLC7A11*, and *SLC3A2*) were down-regulated in breast and lung cancer cell lines upon BRD4 knockdown (57). The inhibition of BRD4 also enhances the expression of a histone deacetylase called sirtuin 1 (SIRT1) (57). Additionally, SIRT1 causes epigenetic reprogramming of epithelial-mesenchymal transition (EMT) thus promotes ferroptosis in head and neck cancer (51).

H2A ubiquitination/de-ubiquitination play a critical role in the regulation of *SLC7A11* expression and erastin-induced ferroptosis (**Figure 1**). PRC1 complex, the best-known ubiquitin ligase of H2Aub, is responsible for the establishment of H2A ubiquitination on *SLC7A11* promoter (58). In contrast, a nuclear deubiquitinating enzyme named BRCA1-associated protein 1 (BAP1) could decrease the H2A ubiquitination occupancy on the *SLC7A11* promoter (59). Interestingly, although H2Aub is generally correlated with gene repression, both BAP1 and PRC1 represses *SLC7A11* expression. The weird results indicate that BAP1 and PRC1 coordinately repress *SLC7A11* expression through dynamic regulation of H2Aub levels on the *SLC7A11* promoter. However, the exact role of H2Aub in *SLC7A11* gene expression requires further research. In addition to H2A, mono-ubiquitination of histone H2B on lysine 120 is an epigenetic active marker associated with *SLC7A11* expression. Wang et al. revealed that P53-mediated repression of SLC7A11 is dependent on USP7-mediated H2B de-ubiquitination (60).

The di- or tri-methylation of H3K9 are well-established epigenetic marks of heterochromatin and associated with transcriptional silencing (61). Inhibition of SUV39H1 (one of histone H3K9me3 methyltransferases) by small chemical molecules or siRNA upregulates *DPP4* expression through reducing the H3K9me3, thereby inducing iron accumulation, lipid peroxidation, and ferroptosis (62). In contrast, KDM3B, a histone H3 lysine 9 demethylase, was reported to prevent erastin-induced ferroptosis of HT-1080 cells (63). Mechanistically, KDM3B knockdown did not change the H3K9 methylation level on the *SLC7A11* promoter, while KDM3B cooperates with transcription factor ATF4 to upregulate the expression of *SLC7A11*. Also, KDM4A, a histone demethylase, was revealed to regulate *SLC7A11* transcription by controlling H3K9me3 demethylation in the promoter of *SLC7A11* (**Figure 1**) (64). Besides H3K9, multiple lysine and arginine residues of histones (such as H3R2, H3K4, H3K27, H3K79, H4R3, H4K20, and H2BK5) also undergo methylation/demethylation dynamics and exhibit pleiotropic roles in gene transcription (65). Therefore, it is of great interest to determine whether the histone methylation of other sites and their writers, readers, and erasers also participate in the regulation of ferroptosis-associated genes.

### RNA Modifications

N6-methyladenosine (m6A) RNA modification emerges in recent years as a new layer of regulatory mechanism controlling gene expression in eukaryotes (66). The m6A RNA modification is a reversible epigenetic modification that targets on mRNA and noncoding RNAs. The m6A modification regulates gene expression by affecting the fate of the modified RNA molecules (67). Intriguingly, m6A modification has been observed to play a regulatory function in ferroptosis. The m6A reader YTH domain containing 2 (YTHDC2) can bind to *SLC7A11* mRNA and thereafter promotes its decay (**Figure 1**) (68). The main death type of tissue ischemic reperfusion injury has been proved to be ferroptosis (11). Xu et al. revealed that m6A methylase methyltransferase like 14 (METTL14) promotes renal ischemic reperfusion injury (69). Mechanistically, they identified *YAP1* mRNA as a target of METTL14 and the translation of m6A-modified *YAP1* mRNA was inhibited. However, a recent study revealed that YAP1 activates *ACSCL4* gene transcription and thereby promotes ferroptosis (69). These paradoxical results suggest that the role of YAP1 in ferroptosis might be context-dependent and tissue or cell-type specific.

### Non-Coding RNAs

The microRNA (miRNA) is a series of single strand noncoding small RNA, which is made up of 20-22 nucleotides. The miRNA can targets the 3’-UTR region of mRNA, triggering mRNA decay or translational inhibition (70). Plenty of miRNAs have been identified to participate in the regulation of the key genes of ferroptosis. For instance, miRNA-17-92 can protect cells from erastin-induced ferroptosis through targeting the ACSL4 axis and down regulating the *ACSL4* expression (71). miRNA-4715-3p induces ferroptosis by inhibiting *GPX4* expression (72). miRNA-137 targets *SLC1A5* to suppress glutamine transportation and induce ferroptosis (73).

The role of long noncoding RNA (lncRNA) in gene regulation is gradually focused during the recent years (74). lncRNA is defined as transcripts of more than 200 nucleotides that are not translated into proteins (75). lncRNAs including HOX transcript antisense RNA and metastasis−associated lung adenocarcinoma transcript 1 are identified in the mechanism of ferroptosis suppress induced by XAV939 treatment (76). The lncRNA P53RRA induces ferroptosis by interacting with Ras GTPase-activating protein-binding protein 1 (G3BP1) and activating p53 pathway, then induce ferroptosis by affecting transcription of several metabolic genes (77). Wang et al. found lncRNA LINC00336 combines with ELAVL1 and inhibit ferroptosis by decreasing intracellular iron and lipid ROS level (78).

Circular RNA (cirRNA) is a type of single-stranded RNA which, unlike linear RNA, forms a covalently closed continuous loop. Circular RNA can regulate gene regulation by directly conjugating mRNA or indirectly transporting miRNAs in the cell (79, 80). In addition, cirRNAs are appealed to involve the ferroptosis regulation. Circular RNA cIARS regulates ferroptosis in HCC cells through interacting with RNA binding protein alkB homolog 5 (ALKBH5) (81). CircABCB10 silencing inhibits the cell ferroptosis and apoptosis by regulating the miR-326/CCL5 axis in rectal cancer (82). Circ_0008035 contributes to cell proliferation and inhibits apoptosis and ferroptosis in gastric cancer *via* miR-599/EIF4A1 axis (83).

Collectively, these findings demonstrate that epigenetic mechanisms contribute to the regulation of ferroptosis-associated genes (**Table 1**). However, whether, when, and how those key regulator genes as well as many newly found genes are epigenetically modulated are poorly understood. Therefore, systematic identification of the epigenetic regulatory network underlying ferroptosis is required in future study.

**Table 1:** Epigenetic regulation of ferroptosis-associated genes.

Type	Molecular mechanism	Consequence on ferroptosis	Reference
DNA modification	Homocysteine treatment inhibits *GPX4* expression through increasing the promoter DNA methylation level	Promotion	(52)
	DNA hypermethylation of *CDH1* increases its expression	Inhibition	(51)
Histone modification	KDM4A induces H3K9me3 demethylation at the promoter region of *SLC7A11* and promotes its transcription	Inhibition	(64)
	BAP1 decreases the H2A ubiquitination level at *SLC7A11* promoter and suppresses its expression	Promotion	(59)
	PRC1 increases the H2A ubiquitination level at *SLC7A11* promoter and suppresses its expression	Promotion	(58)
	USP7 decreases H2Bub1 level at *SLC7A11* promoter and represses its expression	Promotion	(60)
	SUV39H1 modulates the H3K9me3 status of *DPP4* gene promoter and down-regulates its expression	Promotion	(62)
RNA modification	YTHDC2 binds on the mRNA of *SLC7A11* and promotes its decay	Promotion	(68)
	METTL14 deposits m6A on *YAP1* mRNA and inhibits its translation	Promotion	(69)
Noncoding RNAs	miRNA-17-92 down-regulates *ACSL4* expression	Inhibition	(71)
	miRNA-4715-3p inhibits *GPX4* expression	Promotion	(72)
	miRNA-137 suppresses *SLC1A5* expression	Promotion	(73)
	lncRNA P53RRA activates p53 pathways	Promotion	(77)
	lncRNA LINC00336 interacts with *ELAVL1* to decrease the intracellular iron and lipid ROS level	Inhibition	(78)
	cIARS interacts with *ALKBH5*	Promotion	(81)
	CircABCB10 regulates miR-326/CCL5 axis	Inhibition	(82)
	Circ_0008035 regulates miR-599/EIF4A1 axis	Inhibition	(83)

## Targeting Epigenetic Regulation: A New Strategy in the Prevention and Therapy of Ferroptosis-Associated Diseases

As aforementioned, epigenetic mechanisms play a critical role in the regulation of ferroptosis-associated genes, thereby finetuning the cellular response to ferroptotic stress. Therefore, targeting epigenetic regulation represents a promising strategy to enhance or inhibit ferroptosis and has potential application in the prevention and therapy of ferroptosis-associated diseases. Indeed, many epigenetic drugs have been reported to display exciting results in cancer therapy through modulating ferroptosis (**Table 2**).

**Table 2:** Epigenetic drugs that modulate ferroptosis in cancer therapy.

Drug type	Name	Molecular mechanism	Consequence on ferroptosis	Cancer types	Reference
DNMT inhibitors	5-Aza-cd	Inhibition of DNMT by 5-Aza-cd increases the expression of *E-cadherin* and *GPX4*	Inhibition	Head and neck cancer	(51, 52)
BET inhibitors	JQ-1	Inhibition of BRD4 by JQ-1 downregulates the expression of *GPX4, SLC7A11* and *SLCA2*	Promotion	Breast cancer; Lung Adenocarcinoma	(57)
HDAC inhibitors	EX-527	Inhibition of SIRT1 by EX-527 increases EMT	Promotion	Head and neck cancer	(51)
	Vorinostat	Unknown	Promotion	Small cell lung cancer; *IDH1/2* mutant brain tumors	(84)

### DNMT Inhibitors

DNMT inhibitors have been successfully used in the treatment of certain hematopoietic malignancies (85). Moreover, recent work showed that DNMT inhibitors could enhance the efficiency of immune checkpoint inhibitors (ICI) in cancer immunotherapy (86). As aforementioned, 5-Aza-cd treatment could release the DNA methylation-mediated epigenetic silencing of *GPX4* and *CDH1* genes, restoring the resistance of cells to ferroptosis (51, 52). Given that DNMT inhibitors have very broad effect on gene expression and genomic stability, it should be careful to distinguish whether their effect on ferroptosis is achieved through the direct regulation of specific ferroptosis-associated gene or the indirect activation of certain signaling pathways due to epigenetic reprogramming.

### BET Inhibitors

Bromodomain and extra terminal protein (BET) inhibitors are a class of drugs that prevent the interaction between BET proteins and acetylated histones (87–89). Sui et al. revealed that ferroptosis is involved in JQ1-induced cell death of BRCA and LUAD (57). Moreover, treatment with JQ1 and ferroptosis inducers (RSL3, erastin, or sorafenib) produced a satisfactory anticancer effect, suggesting that the combination of BET inhibitors with ferroptosis inducers may become a new therapeutic modality.

### HDAC Inhibitors

Pharmacological inhibition of SIRT1 by EX-527 increases ferroptosis susceptibility by suppressing EMT, while SIRT1 agonists, resveratrol and SRT1720, promote ferroptosis (51). A recent drug screening also identified a class I HDAC inhibitor, Vorinostat, as an inducer of ferroptosis in small cell lung cancer (SCLC) and isocitrate dehydrogenase (*IDH1/2*)-mutant brain tumors, suggesting an unique vulnerability that is regulated by histone or non-histone acetylation (84).

## Discussion

As a new concept introduced in 2012, ferroptosis has attracted tremendous attention in biomedical fields. The existing work about the epigenetic regulation of ferroptosis mainly focused on several key ferroptosis genes. Whether the epigenetic mechanisms affect multiple ferroptosis genes and how these different epigenetic mechanisms corporate with diverse signaling pathways to determine the responsiveness of cells to ferroptosis stimuli remain unknown. Therefore, a systematic study on the epigenetic regulatory network of ferroptosis process is still a blank in this field and requires extensive investigation.

Given that ferroptosis plays a role of surveillance in tumorigenesis and also contributes to the efficiency of multiple cancer therapies [chemotherapy, radiotherapy (90), and immunotherapy (91)], it is rationale to speculate that targeting the epigenetic machines alone or in combination with the traditional therapies will be promising strategies for cancer therapy. Since ferroptosis is closely related to neurodegenerative diseases, ischemia-reperfusion injury of organ, neurotoxicity, and others (11, 92–95), it is also of great interest to explore the epigenetic mechanisms underlying the altered ferroptosis sensitivity under different pathological processes. The advances in this cross-disciplinary research field may shed light on the treatment of diseases mentioned above by modulating ferroptosis.

## Author Contributions

Conceptualization, YP, HW, and LT. Writing—original draft preparation, YP and LT. Writing—review and editing, YQ, HW, and LT. Funding acquisition, LT. All authors have read and agreed to the published version of the manuscript.

## Funding

This review was supported by the National Natural Science Foundation of China (81672785, 31871291, and 82073113) and the National Key R&D Project of China (2016YFA0101800). LT was also supported by the innovative research team of high-level local university in Shanghai.

## Conflict of Interest

The authors declare that the research was conducted in the absence of any commercial or financial relationships that could be construed as a potential conflict of interest.

The reviewer, SJ, declared a shared parent affiliation with the authors to the handling editor at the time of the review.

## Publisher’s Note

All claims expressed in this article are solely those of the authors and do not necessarily represent those of their affiliated organizations, or those of the publisher, the editors and the reviewers. Any product that may be evaluated in this article, or claim that may be made by its manufacturer, is not guaranteed or endorsed by the publisher.
